# Levodopa-induced dyskinesia in Parkinson’s disease: an updated review of pharmacological treatments

**DOI:** 10.3389/fnagi.2025.1684885

**Published:** 2025-10-21

**Authors:** Feifei Chen, Changqing Zhou

**Affiliations:** Department of Neurology, Bishan Hospital of Chongqing Medical University, Chongqing, China

**Keywords:** Parkinson’s disease, levodopa-induced dyskinesia, clinical studies, pharmacological treatments dopaminergic agents, non-dopaminergic medications

## Abstract

Levodopa-induced dyskinesia (LID) remains one of the most disabling complications of long-term dopaminergic therapy in Parkinson’s disease. Despite decades of investigation, only amantadine has been established as the standard FDA-approved treatment, while istradefylline provides a complementary non-dopaminergic option. Most other candidate agents–including memantine, clozapine, and serotonergic or noradrenergic modulators–have shown inconsistent efficacy or safety limitations, underscoring persistent translational challenges between preclinical promise and clinical outcomes. In addition to pharmacological therapies, deep brain stimulation (DBS) serves as an established non-pharmacological intervention for advanced cases. This review systematically synthesizes current pharmacological strategies, consolidating evidence on mechanisms, efficacy, safety, and regulatory status. We further highlight failed or inconclusive trials, emphasize gaps in trial design and patient heterogeneity, and discuss emerging approaches such as individualized therapeutic frameworks, novel drug delivery systems, and AI-assisted drug discovery. Potential complementary pathways, including Traditional Chinese Medicine (TCM), are also briefly noted as alternative directions. By linking mechanistic insights with therapeutic evidence, this review provides an updated framework for optimizing LID management and guiding future research directions.

## 1 Introduction

**FIGURE 1:**
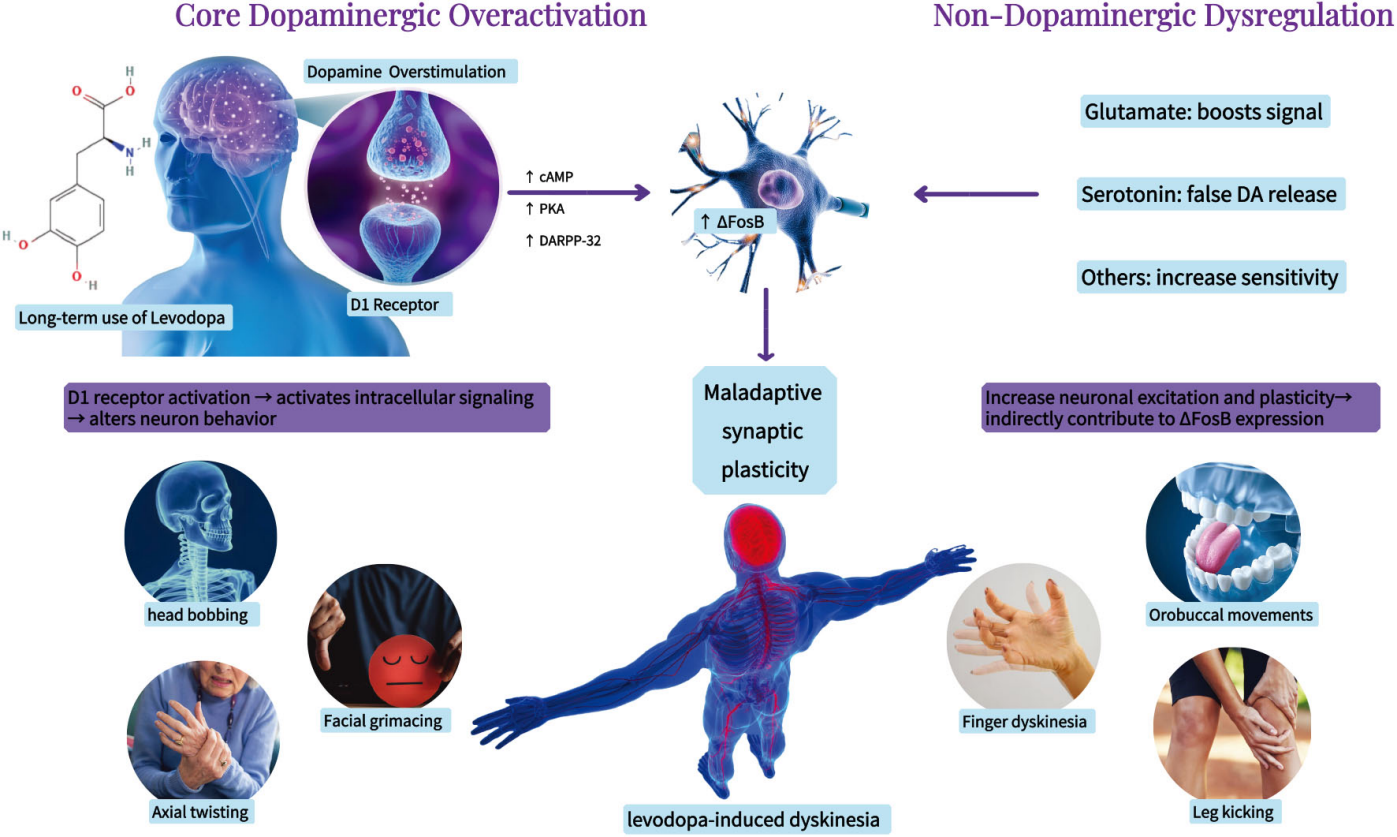
Pathophysiology of levodopa-induced dyskinesia (schematic).

Parkinson’s disease (PD) ranks as the second most prevalent neurodegenerative disorder and is the most rapidly expanding neurological disease globally. The prevalence of PD escalates with age (GBD 2016 Parkinson’s Disease Collaborators, 2018), and due to the swift aging of the global populace and increasing life expectancy, the population of PD sufferers is anticipated to surpass 12 million by 2040 (Dorsey et al., 2018). PD is primarily characterized by motor symptoms such as bradykinesia, resting tremor, stiffness, and abnormalities in posture and gait. It also presents non-motor symptoms like cognitive and psychiatric deficits (Jankovic, 2008). The key feature of PD is the loss of dopaminergic neurons in brain regions associated with movement and the abnormal accumulation of α-synuclein in neurons (Cesaroni et al., 2022). The pathophysiology of PD encompasses various dysfunction pathways, including oxidative stress, mitochondrial dysfunction, disrupted calcium homeostasis, neuroinflammation, and neurotransmitter system dysfunction (Zaman et al., 2021). Currently, no disease-modifying therapy exists, and dopamine replacement with levodopa remains the mainstay of treatment (Antonini et al., 2018). However, with disease progression and long-term levodopa exposure, patients frequently develop levodopa-induced dyskinesia (LID), a motor complication that significantly impairs quality of life (Santos-García et al., 2024). Approximately 25% of patients develop LID after years of levodopa therapy, and the prevalence increases to 80% after ten 5 years (You et al., 2018). LID is characterized by involuntary movements affecting the limbs, trunk, and head, which markedly reduce quality of life (di Biase et al., 2023). Its pathophysiology involves maladaptive synaptic plasticity and abnormal dopaminergic signaling, with additional contributions from multiple non-dopaminergic systems, including glutamatergic, serotonergic, and inflammatory pathways (Calabresi et al., 2010; Espay et al., 2018; Tran et al., 2018) (Figure 1).

In recent years, therapeutic strategies for LID have expanded substantially. Clinical management includes both pharmacological adjustments and surgical interventions such as deep brain stimulation (DBS). DBS has demonstrated remarkable efficacy in alleviating LID symptoms and reducing levodopa dosage requirements (Fasano et al., 2012; Kwon et al., 2022; Li et al., 2021; Munhoz et al., 2014; Toda et al., 2004). However, its invasiveness and limited indications underscore the ongoing need for optimized pharmacological therapies. Therefore, this review provides an updated synthesis of pharmacological treatments for LID, summarizing mechanisms, clinical evidence, efficacy, safety, and regulatory status across drug classes.

## 2 LID evaluation scales

Levodopa-induced dyskinesia significantly impacts patients’ daily activities, quality of life, and overall disability. Therefore, assessing the presence and severity of dyskinesia in patients undergoing levodopa treatment, as well as evaluating treatment efficacy, is of critical importance in disease management. In clinical practice, various rating scales have been developed and utilized for this purpose. Some scales are specifically designed for assessing dyskinesia in PD, while others are components of broader scales used to evaluate overall motor dysfunction in PD. Additionally, certain scales were initially developed for other syndromes associated with dyskinesia and later adapted for PD-related dyskinesia assessment. These scales include the Abnormal Involuntary Movement Scale (AIMS), the fourth part of the Unified Parkinson’s Disease Rating Scale (UPDRS Part IV), items 41 (duration of dyskinesia) and 42 (functional impact of dyskinesia) of the MDS-UPDRS, the Obeso Dyskinesia Rating Scale (CAPIT), the Rush Dyskinesia Rating Scale (RDRS), the Clinical Dyskinesia Rating Scale (CDRS), the Lang-Fahn Activities of Daily Living Dyskinesia Scale (LFADLDS), the Parkinson Disease Dyskinesia Scale (PDYS-26), and the Unified Dyskinesia Rating Scale (UDysRS). Each of these tools provides valuable insights into the severity and functional impact of dyskinesia, contributing to more effective clinical evaluation and management (Colosimo et al., 2010).

## 3 Pharmacological treatment

**FIGURE 2:**
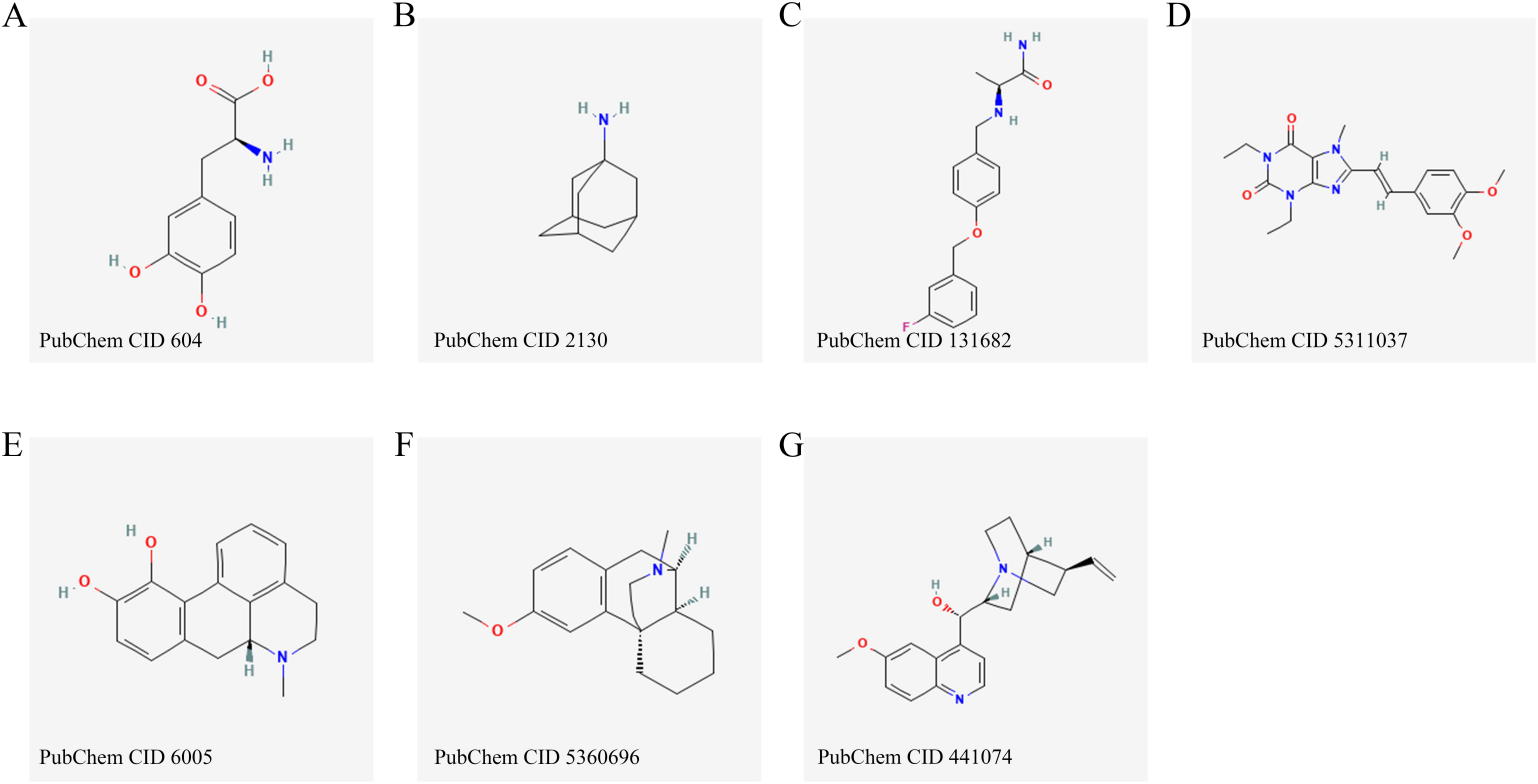
Chemical structures of the main pharmacological agents discussed in this review, including levodopa **(A)**, amantadine **(B)**, safinamide **(C)**, istradefylline **(D)**, apomorphine **(E)**, dextromethorphan **(F)** and quinidine **(G)**.

**FIGURE 3:**
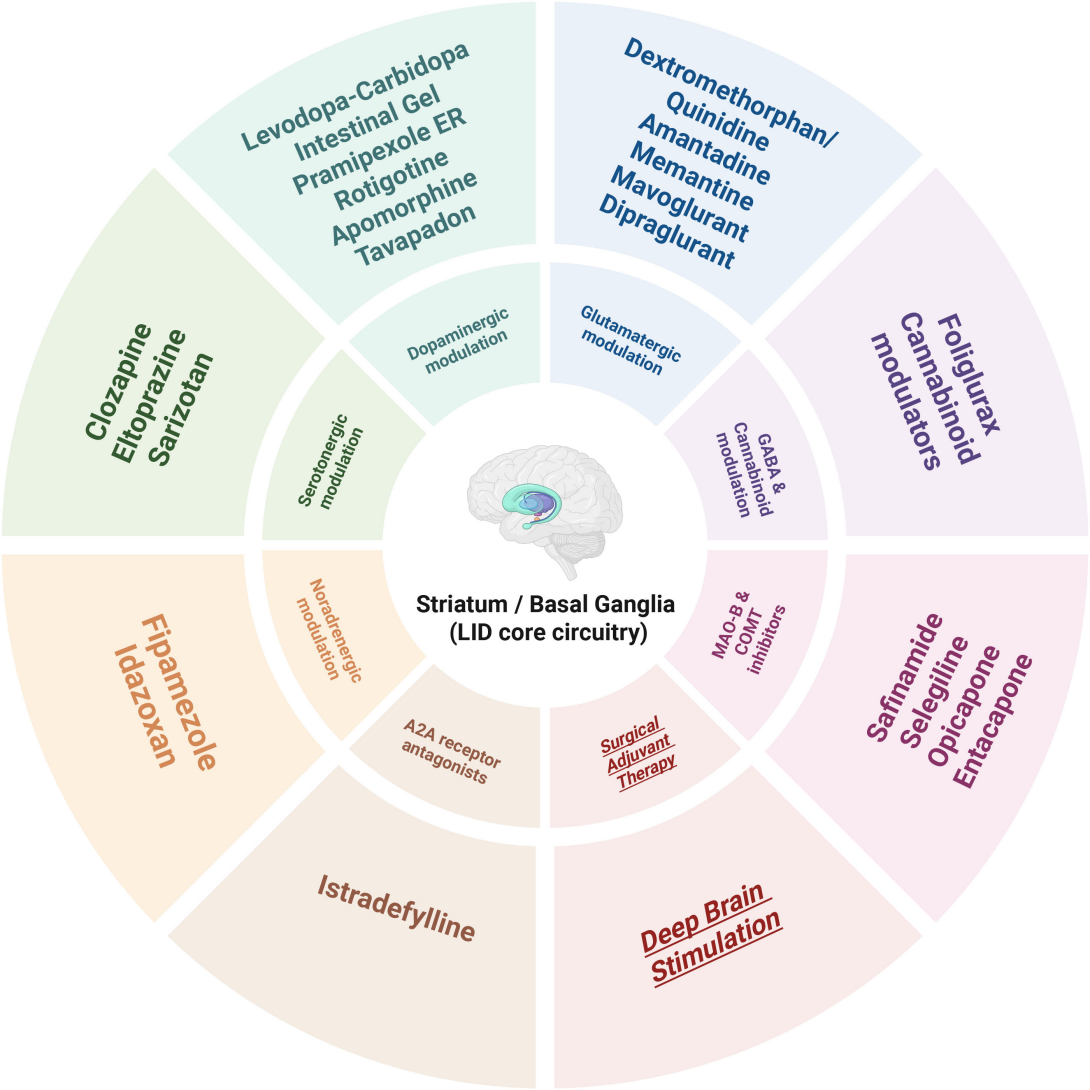
Therapeutic targets and representative drug classes for levodopa-induced dyskinesia (LID).

The chemical structures of the representative agents discussed in this section are summarized in Figure 2 and Supplementary Figure 1. Current pharmacological treatment techniques for LID predominantly emphasize two principal approaches. The primary objective is to attain more consistent dopaminergic activation by creating new levodopa formulations or employing revolutionary drug delivery techniques. The second strategy focuses on non-dopaminergic pathways, utilizing therapies aimed at preventing the onset and progression of dyskinesia. A schematic overview of major therapeutic targets and representative drug classes for LID is shown in Figure 3. A comprehensive summary of pharmacological treatments for levodopa-induced dyskinesia, including mechanisms, clinical outcomes, and trial status, is provided in Table 1.

**TABLE 1:** Pharmacological treatments for levodopa-induced dyskinesia (LID): mechanisms, clinical outcomes, and trial status.

Class	Drug/ formulation	Primary target/ mechanism	Key clinical outcomes (typical measures)	Trial phase/status	Common AEs/notes
Dopaminergic replacement/delivery	LCIG (levodopa–carbidopa intestinal gel)	Continuous jejunal infusion → smoother dopaminergic stimulation	↓ OFF time; mixed effects on LID risk depending on dose optimization; improves motor fluctuations	Approved in many regions	Device-/procedure-related issues (infection, tube problems)
Dopaminergic replacement/delivery	AP09004 (accordion pill levodopa)	Extended GI retention → flatter levodopa PK	Phase II: ↓ OFF without ↑ troublesome dyskinesia reported in early studies	Phase II completed/development ongoing	GI AEs; development status may vary
Dopamine agonists	Pramipexole ER	D2/D3 agonism; once-daily ER profile	In early PD delays motor complications; helps smooth fluctuations	Approved	Somnolence, peripheral edema, impulse-control disorders
Glutamatergic modulation – NMDA	Amantadine IR/ER (ADS-5102)	Non-selective NMDA antagonism; reduces glutamatergic hyperactivity	Robust ↓ UDysRS; ↑ ON time without troublesome dyskinesia; guideline-endorsed symptomatic benefit	FDA-approved (ER for LID)	Hallucinations, peripheral edema, dry mouth; caution in renal impairment
Serotonergic agents	Clozapine (low dose)	5-HT2A/2C antagonism; high-affinity D4 blockade; 5-HT1A agonism	DBPC: ↓ daily duration of LID (∼2 h); ↓ peak LID score in levodopa challenge	Off-label use for LID; efficacy shown in small trials	Risk of agranulocytosis → requires regular CBC monitoring; other antipsychotic AEs
Adenosine A2A receptor antagonists	Istradefylline	Selective A2A receptor blockade on indirect pathway MSNs → reduce ‘stop’ pathway overactivity	↓ OFF by ∼0.7–1.8 h vs. placebo; increases good ON time; may reduce need for levodopa hence indirectly benefit LID	Approved (FDA 2019, PMDA 2013)	Dyskinesia (can occur), insomnia, nervousness; generally tolerable

LCIG, Levodopa-carbidopa intestinal gel; ER, extended-release; IR, immediate-release; UDysRS, Unified Dyskinesia Rating Scale; NMDA, N-methyl-D-aspartate receptor; mGluR5, Metabotropic glutamate receptor 5; AMPA, α-amino-3-hydroxy-5-methyl-4-isoxazolepropionic acid receptor; MAO-B, Monoamine oxidase-B; COMT, Catechol-O-methyltransferase; FDA, U.S. Food and Drug Administration; PMDA, Pharmaceuticals and Medical Devices Agency; DBS, deep brain stimulation; SV2A, synaptic vesicle protein 2A; MSNs, medium spiny neurons; CBC, complete blood count; PK, Pharmacokinetics; GI, Gastrointestinal; AEs, Adverse events; LFT, Liver function test; RCT, randomized controlled trial; PGIC, Patient Global Impression of Change; AUC, area under the curve; h, hours; ON time, period with good motor control; OFF time, period with symptom re-emergence; ↓, decrease/reduction; ↑, increase/elevation; DBPC, Double-blind placebo-controlled.

### 3.1 Dopaminergic agents

#### 3.1.1 Dopamine replacement therapy

Levodopa-induced dyskinesia is significantly linked to the total dosage of levodopa, and clinical practice prioritizes the maintenance of low-dose levodopa regimens to reduce this risk. Levodopa-carbidopa intestinal gel (LCIG), delivered via a continuous infusion device, mimics physiological persistent dopaminergic activation. The method entails diminishing variations in plasma drug concentrations, particularly by reducing the severity and frequency of trough levels, thus simulating the natural control of dopamine neurotransmission (Fernandez et al., 2015). The effect of LCIG on the development of dyskinesia requires personalized evaluation (Antonini et al., 2016). The innovative formulation, AP09004, employs an accordion-like architecture to prolong the absorption duration of levodopa. Phase II clinical trials have shown its efficacy in decreasing OFF time without elevating the occurrence of dyskinesia, suggesting potential therapeutic benefits (LeWitt et al., 2019).

#### 3.1.2 Dopamine agonists

Dopamine agonists are first-line treatments for early-stage PD and have been shown to delay motor complications while reducing LID. Extended-release formulations of pramipexole (Chwieduk and Curran, 2010) and rotigotine transdermal patches (Waters, 2013) further reduce the risk of dyskinesia by maintaining stable plasma drug concentrations. Currently available dopamine agonists include pramipexole, piribedil, ropinirole, rotigotine, and apomorphine. Recent reviews further highlight advances in the therapeutic use of non-ergot dopamine agonists for both motor and non-motor symptoms of PD (Jing et al., 2023). A novel dopamine agonist, tavapadon (Bezard et al., 2024; Moreau et al., 2013), exhibits selective dopamine receptor activation properties and incorporates innovative drug delivery methods. These features may reduce adverse events, improve tolerability, and expand the applicability of dopamine agonists in the treatment of PD.

#### 3.1.3 Continuous dopamine delivery

Apomorphine, a potent dopamine agonist with high affinity for D1 and D2 receptors, has a short half-life and achieves continuous dopaminergic stimulation through subcutaneous infusion via a portable pump. Studies have demonstrated that apomorphine can reduce the occurrence of motor complications while also improving non-motor symptoms such as mood disturbances and hallucinations.

### 3.2 Non-dopaminergic medications

#### 3.2.1 Glutamatergic agents

Glutamate is essential for neural control in the brain via many ionotropic and metabotropic receptors, with the N-methyl-D-aspartate (NMDA) receptor being most closely linked to motor dysfunction.

##### 3.2.1.1 Amantadine

Amantadine, a non-selective NMDA receptor antagonist, is the most prevalent and well-established treatment for LID. Its effectiveness has been consistently corroborated in multiple research. Amantadine was initially suggested as a treatment for PD in 1969, based on an incidental observation of a patient with PD who exhibited symptom improvement after using immediate-release amantadine as an antiviral medicine (Rascol et al., 2022b). Since then, amantadine immediate-release (IR) has been progressively utilized to treat LID and has established itself as a fundamental medication in this therapeutic domain (Elahi et al., 2012; Kong et al., 2017; Verhagen Metman et al., 1998b). Research has shown that amantadine possesses considerable anti-dyskinetic properties in both animal studies and clinical trials. Nevertheless, the application of amantadine IR has specific constraints. The requirement for numerous daily dosages not only elevates the medication burden for patients but also results in significant variations in plasma drug concentrations. Moreover, suboptimal levels of amantadine IR frequently do not yield satisfactory therapeutic results, whilst elevated doses may diminish patient tolerance, so limiting its clinical use (Espay et al., 2018; Fabbrini et al., 2007).

Amantadine has been demonstrated in numerous clinical trials to effectively reduce the severity and duration of LID and decrease daily OFF time in PD patients receiving dopamine replacement therapy. Until recently, it remained the only medication specifically approved by the U.S. FDA for the management of LID. In 2017, a novel extended-release (ER) formulation of amantadine, ADS-5102, received FDA approval, based on clinical trials demonstrating significant reductions in dyskinesia severity, alongside favorable safety and tolerability profiles. Consequently, amantadine ER is currently recommended as a symptomatic treatment for PD patients experiencing LID while on dopaminergic therapies. Additionally, recent clinical practice guidelines, including the 2017 National Institute for Health and Care Excellence (NICE) guidelines and the 2018 International Parkinson and Movement Disorder Society (MDS) guidelines, explicitly endorse the use of amantadine as a primary pharmacological option for managing LID. Recent studies have introduced a novel formulation of amantadine–IR/ER amantadine (OS320)–which combines an IR layer with an ER core. Administered once daily in the morning, this formulation achieves stable plasma drug concentrations throughout the day. The IR layer rapidly releases a portion of the drug within 30–60 min after administration, while the ER core employs an osmotic pump system to sustain drug release, reaching peak plasma concentration approximately 7.5 h post-dose (Rascol et al., 2022b).

The efficacy and safety of OS320 in treating LID were evaluated in two randomized, double-blind, placebo-controlled trials, ALLAY-LID I and ALLAY-LID II. While ALLAY-LID I did not meet its primary endpoint, ALLAY-LID II demonstrated significant therapeutic effects for both the 193 mg and 258 mg dosage groups. Compared to placebo, these groups showed reductions in Unified Dyskinesia Rating Scale (UDysRS) scores of −5.5 and −5.2, respectively. Pooled analyses further revealed that the 258 mg dose significantly increased ON time without troublesome dyskinesia by 1.5 h, primarily through a reduction in ON time with troublesome dyskinesia. In terms of safety, the adverse effects of OS320 were similar to those observed with other amantadine formulations, including hallucinations, dry mouth, nausea, and peripheral edema. While a slightly higher incidence of adverse events was noted at the 258 mg dose, the overall tolerability was favorable, with no new safety concerns identified. An updated pooled analysis of the ALLAY-LID studies further corroborated the effect of OS320 on reducing dyskinesia severity and increasing ON time without troublesome dyskinesia (Rascol et al., 2022b).

##### 3.2.1.2 Memantine

Another NMDA receptor antagonist garnering considerable attention is memantine, which exhibits higher selectivity for NMDA receptors compared to amantadine. Additionally, it exerts stronger antagonistic effects on extrasynaptic receptors, potentially offering more precise regulation of NMDA-dependent glutamatergic hyperactivity in the striatum (Leroi et al., 2009). A 3-week randomized, double-blind, placebo-controlled crossover trial evaluated the efficacy of memantine (20 mg/day) in treating LID. The study included 17 patients, of whom 15 completed the trial (Wictorin and Widner, 2016). Results demonstrated that memantine significantly reduced the duration of dyskinesia, though its effect on dyskinesia severity was limited. Specifically, patient diaries revealed that the duration of dyskinesia decreased from 25% in the placebo group to 16% in the memantine group. However, video-based dyskinesia scores, as assessed by the Clinical Dyskinesia Rating Scale (CDRS), showed no significant differences between groups. Interestingly, the study highlighted notable inter-individual variability in response to memantine. While seven patients exhibited significant improvement in dyskinesia scores (average reduction of 32%), three patients experienced worsening symptoms (average increase of 33%), and five patients showed no significant change. These findings suggest that the therapeutic efficacy of memantine may be closely linked to individual differences. More importantly, this study indicates that, unlike amantadine, memantine appears to primarily reduce the duration of dyskinesia rather than directly alleviating its severity.

Similarly, a 90-day randomized, double-blind, placebo-controlled trial further evaluated the potential efficacy of memantine in addressing axial symptoms and dyskinesia. The study included 25 advanced PD patients, all of whom exhibited severe gait disturbances (Unified Parkinson’s Disease Rating Scale [UPDRS]-III item 29 score ≥2) and pronounced forward-leaning posture (UPDRS-III item 28 score ≥2) (Moreau et al., 2013). The results demonstrated significant improvements in both overall UPDRS motor scores and axial symptom subscores in the memantine group, with reductions of 1.0 and 1.1 points, respectively. Regarding dyskinesia, the memantine group showed significantly greater reductions in Dyskinesia Rating Scale scores and axial subscores compared to the placebo group, with decreases of 1.9 and 1.4, respectively. These findings suggest that memantine may mitigate NMDA receptor overactivation, thereby reducing excessive synaptic noise within the striato-cortical circuitry and improving both the intensity of dyskinesia and axial symptoms. Additionally, memantine was found to significantly alleviate trunk flexion rigidity and improve trunk extensor strength, which may help slow the progression of forward-leaning posture and muscle atrophy in PD patients. However, the study found no significant effects of memantine on gait parameters such as stride length, gait velocity, and cadence, suggesting limited efficacy in addressing gait disturbances. In summary, while this study highlights the potential of memantine in improving axial symptoms and LID, it also underscores challenges, including the small sample size and notable inter-individual variability in treatment responses.

##### 3.2.1.3 Other NMDA/glutamate modulators

Remacemide (RMC) is a non-competitive antagonist of the NMDA receptor with modest affinity. Its active metabolite, AR-R 12495 AR, also demonstrates modest affinity for NMDA receptors and additionally influences voltage-dependent neuronal sodium channels. This dual approach not only exhibits potential for antiepileptic and neuroprotective effects but also mitigates the behavioral and neurotoxic side effects often linked to conventional NMDA receptor antagonists (Schachter and Tarsy, 2000). Research indicates that RMC may have potential benefits in several neurological conditions. In animal models of PD, RMC in conjunction with levodopa has been shown to enhance motor symptoms and shown efficacy in addressing LID. In 1-methyl-4-phenyl-1,2,3,6-tetrahydropyridine (MPTP) -induced primate models of PD, the combination of RMC and levodopa improved Parkinsonian symptoms by 42% (Greenamyre et al., 1994). Moreover, clinical studies have shown that RMC, when used as an adjunct to stable levodopa therapy, does not exacerbate LID and, in certain cases, may improve motor fluctuations. In a multicenter, randomized, double-blind, placebo-controlled dose-ranging study involving 279 patients receiving levodopa treatment, RMC demonstrated good tolerability at doses of 300 mg/day (administered twice daily) or 600 mg/day (administered four times daily). Common adverse events included dizziness, nausea, and drowsiness, but RMC did not worsen LID. Although the study’s statistical power was limited, results indicated a trend toward improvement in ON time and motor UPDRS scores among patients taking RMC at doses of 150 mg/day and 300 mg/day. Notably, in the RMC 150 mg/day group, the average ON time increased by 8.2%, corresponding to an additional 0.84 h per day, without a significant increase in the severity of dyskinesia (Shoulson et al., 2001). In addition, the anti-motor fluctuation effects of RMC became evident by the fourth week of treatment and persisted through the seventh week, suggesting its potential as an effective adjunct to levodopa therapy. The neuroprotective properties of RMC have also been validated in several animal models. For example, it has been shown to reduce infarct volume in ischemic brain injury and mitigate hippocampal neuronal damage, primarily attributed to its NMDA receptor-blocking effects (Palmer et al., 1995). Although current studies indicate that RMC demonstrates good tolerability and potential efficacy in the treatment of LID, research on its application in LID remains limited compared to the more extensive investigations of its use in epilepsy.

Dextromethorphan (DM) is a low-affinity, non-competitive NMDA receptor antagonist with modulatory effects on glutamatergic neurotransmission and potential anti-excitotoxic properties. A small-scale, double-blind, placebo-controlled crossover trial (*n* = 6) demonstrated that, when co-administered with quinidine (to inhibit DM metabolism into dextrorphan), DM reduced the mean and peak LID scores by over 50% without significantly affecting the antiparkinsonian efficacy of levodopa, as no differences in the magnitude or duration of motor improvement were observed (Verhagen Metman et al., 1998a). In some patients, a slight increase in the levodopa threshold dose was noted, suggesting that individualized dose adjustments may be necessary to avoid potential fluctuations in therapeutic effects. Additionally, baseline Parkinsonian symptoms in the OFF state showed mild improvement in the DM group, indicating that DM’s regulation of glutamatergic hyperactivity might have broader effects. These effects may involve mechanisms beyond NMDA receptor antagonism, such as modulation of σ receptors or κ-opioid receptors, which could influence basal ganglia circuitry. The findings from NCT01767129 further support the potential of dextromethorphan/quinidine (DM/Q) in treating LID (Fox et al., 2017). This multicenter, randomized, double-blind, placebo-controlled Phase II trial (*n* = 13) evaluated the effects of DM (45 mg)/Q (10 mg) administered twice daily over a 2-week period. At the conclusion of the treatment, a standardized intravenous levodopa challenge was conducted. Results demonstrated that DM/Q significantly reduced the peak severity score of LID and the AUC for dyskinesia severity from baseline to the end of the ON state. Importantly, DM/Q did not exacerbate Parkinsonian motor symptoms, as indicated by the lack of significant changes in MDS-UPDRS Part III scores. Additionally, patient-reported outcomes further underscored the clinical relevance of DM/Q. Patient Global Impression of Change (PGIC) scores indicated that 69.2% of patients reported significant improvement in LID following DM/Q treatment, compared to only 7.7% in the placebo group. Improvements were also observed in daily living activities, as reflected by the 39-item Parkinson’s Disease Questionnaire (PDQ-39) ADL subscale scores. These findings highlight not only the efficacy of DM/Q in reducing LID severity but also its potential to enhance patients’ quality of life.

However, current studies on DM/Q are limited by small sample sizes and relatively short treatment durations. Future research should focus on larger, long-term trials to confirm these findings. Additional areas of investigation could include exploring lower quinidine doses (e.g., 5 mg twice daily) or sustained-release formulations to balance metabolic inhibition with safety, as well as evaluating the potential synergistic effects of combining DM/Q with amantadine or other glutamate modulators. Such approaches may optimize therapeutic outcomes by targeting multiple pathways associated with LID.

In the previous century, riluzole was recognized as a non-competitive NMDA receptor antagonist that mitigates excitotoxicity by stabilizing the inactivated state of voltage-dependent sodium channels, consequently diminishing presynaptic glutamate release and disrupting the excitotoxic cycle (Doble, 1996). Riluzole has been extensively investigated in preclinical models of many central nervous system illnesses due to its neuroprotective, anticonvulsant, and sedative effects. In models of ischemic cerebral injury, riluzole markedly decreased glutamate release and infarct volume. It has been demonstrated to repair MPTP-induced decreases in striatal dopamine levels and mitigate motor deficits in the 6-hydroxydopamine (6-OHDA) Parkinson’s model. The neuroprotective effects found in animals of several neurodegenerative disorders indicate that riluzole may potentially affect the onset and course of LID. These findings necessitate additional exploration of its therapeutic potential in addressing LID, especially considering its recognized mechanisms of action and preclinical effectiveness in analogous pathological scenarios (Araki et al., 2000; Marin et al., 2000; Pratt et al., 1992). Despite the encouraging results seen in preclinical investigations, the clinical use of riluzole for LID has been constrained by insufficient rigorous clinical trials and variable outcomes. A small, open-label clinical trial yielded initial insights into riluzole’s potential for managing LID. This study involved six patients with advanced PD (3 males and 3 females, aged 49–75 years) who experienced substantial dyskinesias as a result of prolonged levodopa treatment (disease duration: 5–10 years; dyskinesia duration: 3–6 years) (Merims et al., 1999). After a 2-week medication washout period, participants commenced riluzole treatment at an initial dosage of 25 mg per day, which was escalated to 50 mg twice daily for a duration of 4 weeks. Patients and their caregivers recorded the daily duration and intensity of dyskinesias. The findings indicated that riluzole medication markedly decreased the overall length of dyskinesia (average reduction of 24%) and the duration of severe dyskinesia episodes (average reduction of 30%). Significantly, riluzole did not exacerbate Parkinsonian symptoms or reduce the therapeutic effectiveness of levodopa. No significant adverse events were reported, and patients exhibited favorable tolerance to the medication. However, a 3-week double-blind, placebo-controlled study conducted by Bara-Jimenez et al. (2006). raised doubts regarding the clinical efficacy of riluzole. The study enrolled 15 patients with moderate to advanced PD and utilized a dose-escalation protocol (up to 200 mg/day) to evaluate the effects of riluzole on LID. The results demonstrated that, compared to placebo, riluzole neither alleviated the severity of dyskinesia nor influenced the duration of levodopa’s therapeutic effects or the core Parkinsonian symptoms. The researchers speculated that although riluzole may theoretically affect the mechanisms underlying LID by inhibiting glutamate release and blocking NMDA receptors, it might also trigger compensatory mechanisms, potentially interacting with non-ionotropic glutamate receptors, such as metabotropic glutamate receptors (Bara-Jimenez et al., 2006).

Metabotropic glutamate receptor 5 (mGluR5) antagonists, including mavoglurant (AFQ056) (Negida et al., 2021) and dipraglurant (ADX 48621) (Tison et al., 2016), have demonstrated potential in treating levodopa-induced dyskinesia (LID) by reducing hyperactive glutamatergic signaling in the striatum (Tison et al., 2016; Wang et al., 2018). A systematic review and meta-analysis assessed the efficacy of mGluR5 antagonists in improving modified Abnormal Involuntary Movement Scale (mAIMS) scores. The analysis included several RCTs of mavoglurant, revealing that compared to placebo, mavoglurant significantly reduced mAIMS scores, indicating its notable antidyskinetic effects and its ability to alleviate the severity of LID. However, no significant differences were observed in secondary endpoints, such as the impact of dyskinesia on daily living (LFADLDS scores) or UPDRS IV, suggesting that mavoglurant’s clinical benefit may be confined to specific symptom improvements. Further analysis revealed that mavoglurant might be more effective in certain patient subgroups, such as those with moderate-to-severe LID, which may correlate with the degree of hyperactivity in glutamatergic signaling (Wang et al., 2018). Although mavoglurant exhibited an overall favorable safety profile, its clinical development faces challenges, including variability in individual patient responses and uncertainties regarding its long-term efficacy (Negida et al., 2021).

A Phase IIa clinical trial investigated dipraglurant in patients with moderate-to-severe LID. This randomized, double-blind, placebo-controlled, multicenter study enrolled 76 participants, with 52 receiving dipraglurant and 24 assigned to placebo (Tison et al., 2016). The results indicated that dipraglurant, administered at doses ranging from 50 mg to 100 mg three times daily, was generally well-tolerated. Common adverse events included dizziness, nausea, fatigue, and visual disturbances. Only two participants discontinued the trial due to adverse events. In terms of efficacy, dipraglurant significantly reduced peak-dose dyskinesia as measured by mAIMS scores, particularly on Day 1 (50 mg) and Day 14. The AUC for mAIMS scores (0–3 h) also showed significant improvement on Day 14, suggesting that dipraglurant effectively alleviates LID within a short-term timeframe. However, no statistically significant improvement in mAIMS scores was observed on Day 28, which may be attributed to an increased placebo effect or the development of tolerance. Additionally, patients experienced a gradual increase in ON time without dyskinesia, although the difference did not reach statistical significance. Importantly, dipraglurant did not negatively impact the core motor symptoms of PD, as evidenced by stable UPDRS-III scores, nor did it increase OFF time or exacerbate resting tremor, indicating that its antidyskinetic effects were achieved without compromising levodopa’s therapeutic efficacy.

Dipraglurant and mavoglurant both target the mGluR5 pathway and demonstrate potential in alleviating LID, however their efficacy and tolerance profiles vary to a degree. Subsequent research should concentrate on refining dosing protocols, elucidating long-term effectiveness, and investigating their use in varied patient demographics to enhance comprehension of their therapeutic potential.

α-amino-3-hydroxy-5-methyl-4-isoxazolepropionic acid receptor, as key ionotropic receptors mediating glutamate-driven excitatory synaptic transmission, play a critical role in the pathogenesis of LID. Their overactivation is closely associated with enhanced glutamatergic signaling in the cortico-striatal pathway, which exacerbates synaptic plasticity abnormalities within basal ganglia circuits, ultimately contributing to LID development. In animal models of LID, including 6-OHDA-lesioned rats and MPTP-treated non-human primates, increased AMPA receptor expression in the striatum has been observed, along with elevated phosphorylation levels of specific subunits such as GluR1. These molecular changes likely lead to enhanced excitatory synaptic transmission and heightened postsynaptic sensitivity. Moreover, alterations in the molecular and functional properties of AMPA receptors may also influence NMDA receptor activity, further amplifying pathological glutamatergic signaling (Duty, 2012). These findings suggest that dysregulated AMPA receptor activity represents a critical mechanism underlying LID. As such, pharmacological agents targeting AMPA receptors, including antagonists or modulators, may offer a promising therapeutic strategy for mitigating LID.

Perampanel, a non-competitive AMPA receptor antagonist, reduces excitatory glutamate signaling by inhibiting AMPA receptor overactivation. Preclinical studies in animal models have demonstrated the potential of AMPA receptor antagonists in alleviating LID. For instance, LY-300164 (telampanel) and topiramate significantly reduced LID in 6-OHDA-lesioned rats and MPTP-treated non-human primates, while simultaneously enhancing the antiparkinsonian effects of levodopa. These findings provide a theoretical basis for the development of AMPA antagonists as candidate therapies for LID. However, clinical trials based on these encouraging preclinical results have been disappointing. In a randomized, double-blind, placebo-controlled trial, perampanel as an adjunct to levodopa failed to significantly reduce the severity or symptoms of LID in patients (Eggert et al., 2010; Lees et al., 2012). This failure may be attributed to physiological differences between animal models and human PD patients. For example, the role of AMPA receptors might be overemphasized in animal models, while in clinical trials, the complex neurochemical status of patients–shaped by disease progression and prior pharmacological treatments–may diminish the therapeutic efficacy of perampanel. Moreover, the non-selective nature of perampanel may have contributed to its limited clinical efficacy, highlighting the need for the development of next-generation AMPA receptor antagonists with higher selectivity. In particular, selective antagonists targeting calcium-permeable AMPA receptors (Ca-permeable AMPA receptors), such as IEM-1460, have shown promising antidyskinetic effects in preclinical studies. IEM-1460 specifically blocks AMPA receptors lacking the GluR2 subunit, effectively reducing levodopa-induced abnormal involuntary movements (AIMs) and ameliorating molecular abnormalities associated with LID. In both 6-OHDA-lesioned rats and MPTP-treated non-human primates, acute administration of IEM-1460 reduced LID in a dose-dependent manner without impairing motor performance. Furthermore, a 21-day chronic co-treatment regimen demonstrated that IEM-1460 significantly suppressed both the induction and subsequent expression of LID, while also reversing molecular markers related to LID. These findings underscore the potential of selective Ca-permeable AMPA receptor antagonists, such as IEM-1460, as a more targeted and effective therapeutic strategy for managing LID (Kobylecki et al., 2010).

#### 3.2.2 Serotonin agents

5-HT neurons, lacking the negative feedback regulation of dopaminergic terminals, can convert levodopa into DA via aromatic L-amino acid decarboxylase (AADC), resulting in abnormal striatal DA fluctuations and exacerbating the development of LID.

##### 3.2.2.1 Clozapine

Clozapine, an atypical antipsychotic, acts as a 5-HT2A/2C receptor antagonist, with additional high-affinity blockade of D4 receptors and agonism at 5-HT1A receptors, which may collectively contribute to its mechanism of action in alleviating LID. In a double-blind, placebo-controlled study, clozapine administered at an average dose of 39.4 ± 4.5 mg/day significantly reduced the daily duration of LID by approximately 2 h, with improvements becoming evident by the fourth week of treatment (Durif et al., 2004). Additionally, in an acute levodopa challenge test, clozapine significantly reduced the peak LID score at rest (*p* = 0.05), indicating its potential to mitigate the severity of LID. However, its clinical utility is limited by the risk of severe adverse effects, such as agranulocytosis, which necessitates strict monitoring of blood cell counts. This requirement poses a significant barrier to its widespread use in clinical practice.

##### 3.2.2.2 Eltoprazine

The novel 5-HT-targeting drug eltoprazine, a 5-HT1A/1B receptor agonist, reduces levodopa conversion by inhibiting the overactivity of 5-HT neurons (Wang et al., 2019). In animal models of LID, eltoprazine significantly decreases abnormal theta (5–8 Hz) oscillations and restores the directional flow of information between the dorsal striatum (dStr) and the substantia nigra pars reticulata (SNr), thereby alleviating dyskinesia symptoms (Wang et al., 2019). In a study using the 6-OHDA dopamine-depleted animal model, chronic levodopa administration was found to significantly enhance theta-band (5–8 Hz) oscillations in the dStr and the SNr. These oscillations were positively correlated with AIM scores. Granger causality analysis revealed that the directionality of theta oscillatory information flow was primarily from the dStr to the SNr, indicating that striatal activity plays a dominant role in the pathophysiology of LID. Long-term administration of eltoprazine significantly reduced theta oscillatory activity in both the dStr and SNr and decreased theta-band information flow between these regions, ultimately alleviating LID symptoms. Importantly, eltoprazine’s antidyskinetic effects did not compromise the motor improvements mediated by levodopa, highlighting its significant advantage as a potential treatment for LID. This characteristic is attributed to eltoprazine’s mechanism of action, which involves modulating 5-HT1A/1B receptors to inhibit aberrant dopamine release from 5-HT neurons, rather than directly interfering with the dopaminergic signaling pathway. In a double-blind, randomized, placebo-controlled Phase I/IIa dose-finding study, 22 LID patients were treated with eltoprazine at doses of 2.5 mg, 5 mg, and 7.5 mg, or placebo. The results demonstrated that 5 mg of eltoprazine significantly reduced the AUC of the CDRS by 1.02 and lowered the maximum CDRS score by 1.14, without compromising the antiparkinsonian efficacy of levodopa (Svenningsson et al., 2015). Furthermore, studies have shown that eltoprazine not only significantly reduces the severity of LID but also holds potential for its prevention and long-term management (Pinna et al., 2016). The adverse effects of eltoprazine are primarily mild, including nausea and dizziness, which tend to diminish with repeated administration. Notably, no significant anxiety or depressive symptoms have been observed.

##### 3.2.2.3 Sarizotan

Sarizotan is a drug with 5-HT1A receptor agonist activity and high affinity for the D2 receptor family (D3 > D4 > D2). Due to its relatively low affinity for other receptors, such as 5-HT2A, 5-HT2C, and 5-HT3, as well as ion channels, sarizotan exhibits high selectivity. It is considered a promising candidate for alleviating LID symptoms by modulating serotonergic and dopaminergic signaling pathways. In a randomized, double-blind, placebo-controlled Phase IIb multicenter study, 398 PD patients were randomized to receive sarizotan at doses of 2 mg/day, 4 mg/day, 10 mg/day, or placebo. The primary endpoint was the change in “on time without dyskinesia” duration (Goetz et al., 2007). The results indicated that while sarizotan at certain doses (e.g., 2 mg/day) demonstrated significant improvements in the duration of dyskinesia and disability scores on Part IV of the Unified PD Rating Scale (UPDRS IV items 32 and 33), it did not significantly outperform placebo in improving the primary endpoint. Additionally, high-dose sarizotan (10 mg/day) was significantly associated with an increase in “off” time and mild adverse events, including nausea, drowsiness, and falls. However, the treatment was generally well-tolerated. Further mechanistic studies revealed sarizotan’s unique pharmacological profile, acting as a full agonist at 5-HT1A receptors and as an antagonist or partial agonist at D2 receptor family subtypes (Bartoszyk et al., 2004). Studies have shown that sarizotan significantly reduces the accumulation of the serotonin precursor 5-HTP and the release of DA, while simultaneously increasing the levels of DA metabolites, including DOPAC and HVA. These findings suggest that sarizotan exerts dual regulatory effects on serotonergic and dopaminergic signaling pathways. Additionally, sarizotan exhibits antidyskinetic effects in animal models, including the suppression of LID and neuroleptic-induced tardive dyskinesia, which are attributed to its 5-HT1A receptor agonist activity (Altwal et al., 2021). Its antidyskinetic effects may also involve partial antagonism of D3 and D4 receptors, which have been implicated as potential targets in dyskinesia research. Furthermore, sarizotan’s prominent 5-HT1A agonist activity supports its anticataleptic effects (Gerlach et al., 2011). Other serotonergic strategies, such as the highly selective 5-HT1A agonist NLX-112, have recently reported Phase 2a clinical trial data, highlighting ongoing interest in this pathway (Svenningsson et al., 2025). In summary, the unique molecular mechanism of sarizotan, along with its potential to alleviate LID symptoms, positions it as a promising candidate for the treatment of PD-associated motor complications.

#### 3.2.3 GABAergic agents

Metabotropic glutamate receptor 4 (mGluR4) is a Gi/o protein-coupled receptor widely distributed at the presynaptic terminals of the basal ganglia and cerebellum. Its activation modulates the excitatory/inhibitory balance of the direct and indirect pathways in the basal ganglia by inhibiting the release of γ-aminobutyric acid (GABA) and glutamate. This mechanism can alleviate motor symptoms of PD. Positive allosteric modulators (PAMs) of mGluR4 represent a promising non-dopaminergic therapeutic approach, offering potential benefits for addressing the unmet needs of PD patients experiencing OFF periods (Charvin et al., 2018b; Duty, 2012). Preclinical studies have demonstrated that PAMs of mGluR4, such as Foliglurax, can reduce motor impairments and alleviate LID in primate models of PD induced by MPTP (Charvin et al., 2018a). Building on this mechanism, a 28-day randomized, double-blind, placebo-controlled Phase II clinical trial evaluated the efficacy and safety of Foliglurax, the first clinical mGluR4 PAM developed for the symptomatic treatment of PD, in 157 patients with motor complications (Rascol et al., 2022a). The results indicated that while the Foliglurax treatment groups (10 mg or 30 mg, twice daily) exhibited a dose-dependent reduction in daily awake OFF time (a decrease of 0.55 h in the 10 mg group and 0.72 h in the 30 mg group, compared to a 0.29-h reduction in the placebo group), the primary endpoint–change in OFF time at day 28–did not achieve statistical significance compared to placebo. Similarly, secondary endpoints, including the severity of dyskinesia and UDysRS scores also failed to show significant improvements. Foliglurax was generally well-tolerated, with no major safety concerns identified. However, treatment-related adverse events, such as falls and proteinuria, were reported at slightly higher rates in the Foliglurax groups compared to placebo. These findings suggest that Foliglurax did not provide significant improvements in motor complications for PD patients, raising challenges to the therapeutic hypothesis targeting the glutamatergic system, specifically mGluR4. This may reflect the complexity of mGluR4 regulatory mechanisms in human PD or highlight discrepancies between preclinical models and clinical efficacy in humans.

Cannabinoid-based therapies have also been explored. The endocannabinoid system plays a significant role in modulating basal ganglia circuitry through CB1 and CB2 receptors, which are implicated in the pathophysiology of LID. Cannabinoid receptors are densely concentrated in the basal ganglia, and their activation enhances GABAergic transmission in the external segment of the globus pallidus (GPe) while reducing glutamate release in the striatum. Specifically, stimulating cannabinoid receptors in the internal segment of the globus pallidus (GPi) reduces GABA reuptake, thereby enhancing GABAergic transmission (Pisani et al., 2011). Cannabinoid receptor agonists may alleviate LID by attenuating excessive fluctuations in striatal dopaminergic signaling and mitigating excitotoxicity associated with glutamatergic overactivity. Conversely, cannabinoid receptor antagonists may improve symptoms by blocking abnormal synaptic plasticity. A study investigating cannabinoid levels in PD patients with and without dyskinesia suggested that the endocannabinoid system is involved in the pathophysiology of PD symptoms. However, the precise role of the endocannabinoid system in the pathophysiology of LID remains unclear (Marchioni et al., 2020).

#### 3.2.4 Noradrenergic agents

The action mechanism of the α-2A adrenergic receptor antagonist fipamezole involves the inhibition of presynaptic α-2 adrenergic receptors, hence augmenting norepinephrine release and influencing basal ganglia circuits. In non-human primates with MPTP-induced lesions, fipamezole markedly diminished the incidence of LID while augmenting the antiparkinsonian effects of levodopa (Savola et al., 2003). An early small-scale (*n* = 10) double-blind studies have demonstrated that single doses of 60 mg and 90 mg fipamezole were effective in reducing the severity of LID in patients with PD (Lewitt et al., 2012). A study involving 179 patients receiving stable levodopa therapy (115 from the United States and 64 from India) evaluated the effects of fipamezole at doses of 30 mg, 60 mg, and 90 mg three times daily over a 4-week period. The results showed that, in the overall population, the primary endpoint–changes in the LIDS score–did not reach statistical significance. However, significant heterogeneity between the U.S. and Indian cohorts was observed, with the U.S. group having a longer disease duration (median of 12 years vs. 8.3 years since PD diagnosis) and a longer duration of levodopa use (10.5 years vs. 7.3 years). A pre-specified subgroup analysis revealed that in U.S. patients, treatment with 90 mg of fipamezole for 28 days led to a significant improvement in LIDS scores compared to placebo (mean difference: −1.9), with a dose-dependent trend of improvement. Importantly, this dose did not worsen Parkinsonian motor symptoms, as evidenced by the lack of significant changes in UPDRS-III scores. Additionally, fipamezole was well-tolerated, with the most common adverse events being transient mild blood pressure elevation, nausea, and taste disturbances. The incidence of serious adverse events was comparable between the fipamezole and placebo groups. These findings suggest that fipamezole may exert its therapeutic effects by stabilizing dopaminergic fluctuations. However, further studies are needed to confirm its efficacy and consistency across diverse patient populations.

Idazoxan, another α-2A adrenergic receptor antagonist, has shown potential in preclinical studies to reduce dyskinesia by modulating the indirect pathway and alleviating the excessive inhibition of the subthalamic nucleus by the globus pallidus. This mechanism may underlie its antidyskinetic effects, as demonstrated in MPTP-lesioned primate models of PD, where idazoxan reduced dyskinesia and enhanced the antiparkinsonian effects of levodopa (Henry et al., 1999). However, clinical trials have not provided definitive evidence for its efficacy in LID. In a randomized, placebo-controlled, 3-week clinical trial, oral administration of idazoxan (20 mg, three times daily) failed to produce significant improvements in LID. Among the eight participants enrolled, only four completed the final analysis, and neither video-based scoring nor patient diaries demonstrated statistically significant differences between idazoxan and placebo. The study suggested that the lack of efficacy might be attributed to suboptimal dosing, as the effective dose in animal studies reached up to 10 mg/kg, whereas the average dose in the human trial was only 0.35 mg/kg. Additionally, the use of apomorphine rather than levodopa for acute challenge testing in the trial may have influenced the results. Notably, higher doses of idazoxan (e.g., a single dose of 40 mg) might paradoxically exacerbate dyskinesia, raising concerns about potential dose-dependent effects. Further studies are needed to clarify the therapeutic window and clinical relevance of idazoxan in the management of LID. In addition, idazoxan demonstrated a high incidence of adverse effects, including flushing (83%), headache (50%), nausea (75%), and vomiting (38%), which led to the premature withdrawal of three patients from the trial due to side effects. The frequency of these adverse reactions was significantly higher compared to the placebo group. These safety concerns severely limit the clinical utility of idazoxan, particularly in PD patients who often experience pre-existing autonomic dysfunction, such as orthostatic hypotension. The potential for idazoxan and related compounds to exacerbate these symptoms further diminishes their feasibility as therapeutic options for managing LID.

### 3.3 Other therapies

#### 3.3.1 Levetiracetam

Levetiracetam (LEV), an antiepileptic drug with long-standing use in Western countries, acts by modulating synaptic vesicle protein 2A (SV2A) to inhibit excessive glutamate release and regulate calcium channel activity. Its potential value in the treatment of LID is attributed to its ability to modulate abnormal neuronal synchronization in the basal ganglia. Preclinical studies in animal models have demonstrated that LEV can regulate the “priming phenomenon,” which is associated with long-term synaptic plasticity, suggesting its therapeutic potential for LID management (Bezard et al., 2004; Hill et al., 2004). However, clinical research on LEV has yielded significantly heterogeneous results. A systematic review of seven trials (*n* = 150) indicated that only three randomized controlled trials demonstrated a mild antidyskinetic effect of LEV, with small effect sizes observed in improvements in UPDRS IV and AIMS scores. In contrast, four open-label studies were prematurely terminated due to poor tolerability–characterized by a high incidence of adverse effects such as dizziness and drowsiness (occurring in >30% of participants)–or insufficient efficacy. The review ultimately concluded that current evidence does not support the clinical effectiveness of LEV in the management of LID (Ebada et al., 2019).

In contrast to earlier findings, the VALID-PD study–the largest double-blind RCT in this field to date–demonstrated that LEV at a dose of 1,000 mg/day, administered with gradual titration, significantly improved LID. Patient diaries indicated a 75-min reduction in on time with dyskinesia (−7.85%), while the dyskinesia duration assessed by UPDRS IV item 32 was reduced by 0.35. Additionally, the CGI scale showed an improvement of 0.7, and no increase in OFF time was observed (Stathis et al., 2011). The discrepancy between VALID-PD and earlier studies may be attributed to differences in dosing strategies. The VALID-PD trial employed slow titration (starting at 250 mg and increasing over 4 weeks to a maintenance dose of 1,000 mg/day) and maintained a moderate dosing regimen. In contrast, many trials included in the systematic review used rapid titration or higher doses (2,000–3,000 mg/day), which likely contributed to poor tolerability and higher dropout rates (VALID-PD: 2.6% dropout rate vs. up to 25% in the systematic review). It is important to note the limitations of the VALID-PD study, including its crossover design being converted to a parallel-group design, which reduced the sample size to 38 participants. Nonetheless, its rigorous double-blind, multicenter methodology provides the highest level of evidence currently available. Future large-scale Phase III trials are needed to confirm the efficacy and long-term safety of LEV using a slow-titration protocol at moderate doses (1,000 mg/day).

#### 3.3.2 Monoamine oxidase-B (MAO-B) inhibitors

Monoamine oxidase-B inhibitors reduce dopamine degradation by inhibiting monoamine oxidase-B while also exerting non-dopaminergic effects, such as modulating glutamatergic transmission and ion channel activity, making them a crucial strategy for the treatment of LID. Safinamide, an MAO-B inhibitor approved by the U.S. FDA in 2017, not only inhibits MAO-B but also enhances dopamine reuptake, promotes glutamate release, blocks voltage-dependent sodium channels, and regulates calcium channels, thereby reducing the occurrence of dyskinesia. Binde et al. (2018) conducted a meta-analysis of 27 clinical studies and confirmed the efficacy of MAO-B inhibitors both as monotherapy and in combination with levodopa. The results highlighted the advantages of selegiline in reducing levodopa dosage and delaying the onset of dyskinesia. These findings support the role of MAO-B inhibitors as an important therapeutic option in improving motor complications associated with PD.

#### 3.3.3 Catechol-O-Methyltransferase (COMT) Inhibitors

Catechol-O-methyltransferase inhibitors, including entacapone, tolcapone, and opicapone, function by inhibiting the peripheral and central metabolism of levodopa, thereby prolonging its half-life. This mechanism stabilizes dopaminergic stimulation and reduces the pulsatile receptor activation associated with LID. Among these, the third-generation COMT inhibitor opicapone has gained attention in recent years due to its long-acting properties and favorable safety profile. Opicapone’s efficacy and tolerability have been demonstrated in data from 33 clinical studies involving over 1,000 PD patients. Results from two Phase III trials (Ferreira et al., 2018) revealed that, compared to placebo, opicapone significantly reduced OFF time while extending “on time” without increasing motor complications. Furthermore, these benefits were sustained for nearly 1 year, supporting its potential as a valuable therapeutic option for managing motor fluctuations in PD.

#### 3.3.4 Adenosine A2A receptor antagonist

The adenosine A2A receptor is a crucial non-dopaminergic regulatory target in the central nervous system, predominantly expressed on the interneurons of the basal ganglia striatum, particularly on medium spiny neurons (MSNs) responsible for inhibitory functions. In PD, the degenerative damage to the nigrostriatal dopaminergic system leads to a progressive upregulation of A2A receptors, which exhibit basal activity. This upregulation exacerbates the hyperactivity of the inhibitory pathway, further contributing to bradykinesia. The excessive activation of A2A receptors has been metaphorically described as an “emergency brake.” Even though dopamine replacement therapies, such as levodopa, act as an “accelerator” by activating the direct “go” pathway, the abnormal activity of A2A receptors continues to hinder the improvement of motor function. This highlights the importance of targeting A2A receptors to overcome the limitations of dopaminergic treatments in managing PD-related motor symptoms (Fuxe et al., 2007; Schiffmann et al., 2007).

Istradefylline is a selective adenosine A2A receptor antagonist with high affinity and specificity. By blocking A2A receptor activity, it effectively suppresses the hyperactivity of the indirect “stop” pathway, thereby “releasing the brake.” Istradefylline was first approved in Japan in 2013 for use in PD patients and, in 2019, became the only non-dopaminergic therapy approved by the FDA for the treatment of OFF time during levodopa combination therapy. The efficacy and safety of istradefylline have been validated in multiple randomized, double-blind, placebo-controlled clinical trials. The FDA’s approval was based on the results of four pivotal trials–6002-US-005, 6002-US-013, 6002-0608, and 6002-009–which included patients with moderate-to-advanced PD (Hoehn and Yahr stages 2–4). All participants were receiving levodopa therapy but experienced OFF time symptoms lasting ≥2–3 h per day. These trials collectively established istradefylline as a significant adjunctive treatment option for managing motor fluctuations in PD (Hauser et al., 2008; Isaacson et al., 2022; LeWitt et al., 2008). These results demonstrated that istradefylline (20 or 40 mg/day) significantly reduced OFF time by 0.7–1.8 h compared to placebo and notably increased good ON time. The incidence of adverse events was similar to that of the placebo group, indicating a high level of safety and tolerability. Furthermore, as istradefylline’s mechanism of action is independent of the dopaminergic system, it may serve as an important adjunctive therapy in patients with troublesome dyskinesia or poor tolerance to dopaminergic treatments. Overall, istradefylline offers a novel therapeutic option for managing motor complications in PD through its distinct non-dopaminergic mechanism, providing an innovative approach to optimizing treatment strategies.

### 3.4 Impact of DBS on pharmacological strategies for LID

Deep brain stimulation is a well-established therapy for managing motor complications in PD and has been validated in millions of patients worldwide (Rowland et al., 2017; Zibetti et al., 2011). By directly modulating basal ganglia activity, DBS reduces the dependence on high-dose levodopa. Studies have shown that subthalamic nucleus (STN)-DBS can reduce levodopa-equivalent daily dose (LEDD) by 30%–50%, which is widely recognized as one of the key benefits of this surgical intervention (Østergaard and Aa Sunde, 2006; Thobois et al., 2013; Zibetti et al., 2011). This decreased reliance on levodopa not only lowers the risk of LID but also reduces the incidence of dose-related side effects such as hallucinations, sedation, and orthostatic hypotension (Thobois et al., 2013; Vizcarra et al., 2019).

Moreover, the combination of DBS and levodopa has demonstrated a synergistic effect, resulting in greater motor improvement than either treatment alone. Meta-analyses have revealed that the combined Stimulation-ON/Medication-ON condition achieves significantly better motor outcomes (as assessed by UPDRS-III scores) compared to Stimulation-ON/Medication-OFF or Stimulation-OFF/Medication-ON conditions, with clinically meaningful benefits maintained beyond 5 years (Okun et al., 2014; Vizcarra et al., 2019). However, aggressively reducing LEDD following DBS may not always be advantageous. While lowering dopaminergic medications can mitigate dyskinesia duration, excessive reductions may lead to apathy, depression, and a diminished synergistic effect between DBS and levodopa (Okun et al., 2014; Rowland et al., 2017). Therefore, postoperative management should focus on balancing LEDD reductions with preserving the additive motor benefits provided by DBS and levodopa.

In addition, pallidal deep brain stimulation (GPi-DBS) has proven to be effective in treating levodopa-induced biphasic-like dyskinesia, offering life-changing benefits for patients refractory to medical management (Okun et al., 2014; Vizcarra et al., 2019). For patients with complex or refractory LID, DBS serves as a complementary approach to stabilize motor fluctuations and enhance the efficacy of pharmacological interventions. Despite its advantages, DBS does not entirely replace the foundational role of pharmacological treatments, which remain crucial for non-surgical candidates and for addressing symptoms beyond the therapeutic scope of DBS. Therefore, integrating DBS with optimized pharmacological therapies represents a promising direction for personalized LID management.

## 4 Discussion

As a major clinical challenge in the management of neurodegenerative diseases, PD-associated dyskinesia is essentially a motor complication induced by long-term levodopa therapy. Its high prevalence and debilitating nature significantly affect patients’ quality of life. Moreover, insufficient understanding of the pathophysiology of LID and its correlation with dopaminergic drug dosing exacerbates the vicious cycle of reduced treatment adherence and symptom progression.

In terms of pharmacological therapy, only amantadine remains the established standard and the first FDA-approved drug for the management of LID, while istradefylline provides complementary benefit by targeting the adenosine A2A pathway. Other agents such as memantine, clozapine, eltoprazine, and fipamezole have shown promising antidyskinetic signals in early studies but remain limited by small sample sizes, inconsistent efficacy, or safety concerns. This highlights that despite decades of research, most non-dopaminergic interventions have yet to demonstrate robust and reproducible clinical benefit.

Importantly, regional and global differences in drug approvals and accessibility add further complexity to clinical practice. Amantadine immediate-release is widely available worldwide, whereas its extended-release formulation (ADS-5102) is FDA-approved but not accessible in many countries. Similarly, istradefylline was first approved in Japan in 2013 and later by the U.S. FDA in 2019, yet it remains unavailable in Europe. These disparities reflect divergent regulatory pathways and evidence thresholds, creating heterogeneity in treatment strategies. As a result, clinicians in some regions must rely on older agents or off-label prescribing, which limits equitable access to newer therapies and underscores the importance of harmonizing international drug approval processes.

More broadly, the translational gap persists: preclinical studies of glutamatergic and serotonergic modulators frequently yield encouraging results, yet clinical trials often fail to reproduce efficacy or are hampered by adverse effects. These discrepancies may reflect fundamental differences between experimental disease models and the complex pathophysiology of human PD, as well as heterogeneity in patient populations. Recent landscape reviews (2024–2025) summarizing ongoing and completed LID clinical trials further underscore this translational gap and highlight the heterogeneity of trial endpoints (Al-Kassmy et al., 2025; Alsalmi et al., 2024).

Critical lessons can also be drawn from failed or inconclusive clinical trials. For instance, glutamatergic modulators such as riluzole and AMPA receptor antagonists (e.g., perampanel) demonstrated strong antidyskinetic effects in preclinical models but produced negative or inconclusive outcomes in randomized controlled trials, underscoring the limitations of animal-to-human translation. Serotonergic agents such as clozapine and sarizotan showed antidyskinetic potential, yet their use was constrained by agranulocytosis risk or paradoxical worsening of OFF symptoms. Trials of memantine and dextromethorphan/quinidine were hindered by very small sample sizes and short treatment durations, likely underestimating therapeutic benefit or failing to detect delayed adverse events. Moreover, the dipraglurant program illustrates how placebo effects and inter-individual variability can obscure drug effects, with early antidyskinetic improvements not sustained at later timepoints. Together, these cases highlight that translational barriers arise not only from biological complexity but also from trial design, endpoint selection, and safety considerations.

In addition to pharmacological strategies, non-pharmacological and complementary approaches warrant consideration. Deep brain stimulation (DBS), particularly targeting the subthalamic nucleus (STN) or globus pallidus interna (GPi), has consistently demonstrated robust efficacy in reducing levodopa dose requirements and alleviating dyskinesia in advanced cases. Although its invasiveness and limited eligibility criteria restrict widespread use, DBS remains an established adjunctive therapy that complements pharmacological management (Lv et al., 2018). Furthermore, in some regions, Traditional Chinese Medicine (TCM) and herbal formulations are being explored as alternative or adjunctive strategies. While current evidence is preliminary and lacks large-scale randomized controlled trials, early findings suggest potential neuroprotective and anti-dyskinetic effects (Liu and Wang, 2024). Incorporating such approaches into rigorous clinical research frameworks may help clarify their role in the broader therapeutic landscape of LID.

Looking forward, overcoming these barriers will require a multidimensional research strategy. First, the development of dynamic monitoring technologies to enable more precise dopaminergic drug delivery should be prioritized. Second, advancing individualized therapeutic frameworks based on pathophysiological subtypes may allow for more targeted interventions. In parallel, emerging platforms such as organoid disease models and artificial intelligence–driven drug screening may accelerate the identification of more selective neuromodulatory agents.

Ultimately, effective control and prevention of LID will require bidirectional translational efforts bridging basic research and clinical practice. Moreover, recent network meta-analyses comparing multiple anti-dyskinetic drugs provide an updated quantitative framework for positioning emerging therapies (Chen et al., 2025). By integrating mechanistic insights, innovative pharmacology, and personalized treatment paradigms, future strategies hold promise for alleviating this disabling complication of Parkinson’s disease.

## 5 Conclusion

Despite decades of investigation, levodopa-induced dyskinesia remains a major unmet challenge in Parkinson’s disease. Amantadine is still the only firmly established pharmacological option, while most non-dopaminergic strategies have shown limited or inconsistent efficacy. Several critical gaps persist: the lack of reliable biomarkers to predict LID onset or treatment response, insufficient large-scale multicenter trials to validate promising candidates such as serotonergic and glutamatergic modulators, and limited understanding of patient-specific pathophysiological subtypes. In addition, regional disparities in drug approval and access continue to restrict equitable care.

Future research should prioritize (1) biomarker discovery for early identification and stratification of patients at risk; (2) harmonized, multicenter clinical trials of emerging therapies with standardized endpoints; and (3) integrative approaches combining precision pharmacology with advanced drug delivery technologies. Addressing these priorities will be essential to bridge the translational gap and move toward effective, personalized management of LID in Parkinson’s disease.

## Supplementary material

Supplementary Figure 1 Chemical structures of additional pharmacological agents discussed in this review, including riluzole **(A)**, selegiline **(B)**, rasagiline **(C)**, entacapone **(D)**, tolcapone **(E)**, opicapone **(F)**, pramipexole **(G)**, ropinirole **(H)**, clozapine **(I)**, and fluoxetine [**(J)**, as a representative SSRI].
