# Supplementary figures and images for: Levodopa-induced dyskinesia in Parkinson’s disease: an updated review of pharmacological treatments
Source: Front Aging Neurosci. 2025 Oct 21;17:1684885. doi: 10.3389/fnagi.2025.1684885 (PMC12583070; doi:10.3389/fnagi.2025.1684885)

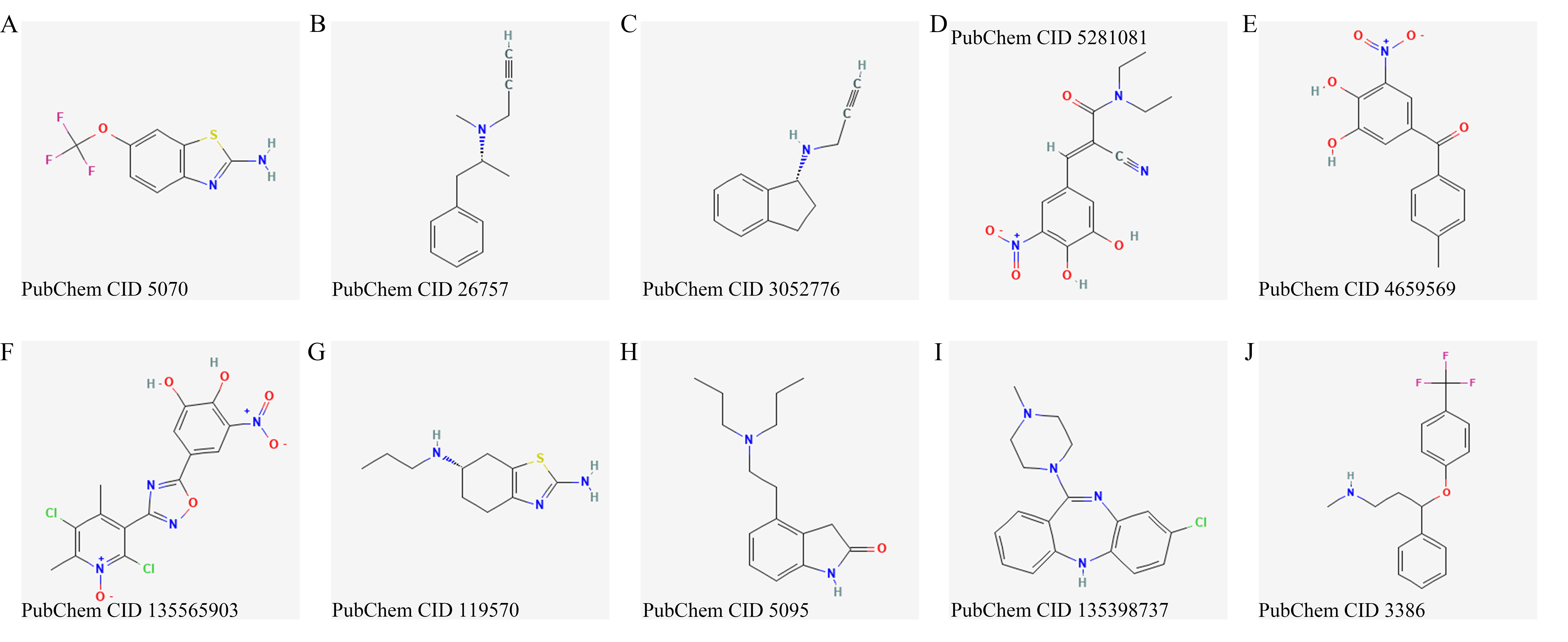

Supplement: Supplementary Figure 1 — Chemical structures of additional pharmacological agents discussed in this review, including riluzole (A), selegiline (B), rasagiline (C), entacapone (D), tolcapone (E), opicapone (F), pramipexole (G), ropinirole (H), clozapine (I), and fluoxetine [(J), as a representative SSRI]. [file Image_1.tif]
